# Detergent-Free Functionalization of Hybrid Vesicles with Membrane Proteins Using SMALPs

**DOI:** 10.1021/acs.macromol.2c00326

**Published:** 2022-04-21

**Authors:** Rosa Catania, Jonathan Machin, Michael Rappolt, Stephen P. Muench, Paul A. Beales, Lars J. C. Jeuken

**Affiliations:** † Astbury Centre of Structural Molecular Biology, University of Leeds, Leeds LS2 9JT, U.K.; ‡ School of Biomedical Sciences, University of Leeds, Leeds LS2 9JT, U.K.; § School of Food Science and Nutrition, University of Leeds, Leeds LS2 9JT, U.K.; ∧ School of Chemistry, University of Leeds, Leeds LS2 9JT, U.K.; ∥ Leiden Institute of Chemistry, University Leiden, Leiden 2300RA, The Netherlands

## Abstract

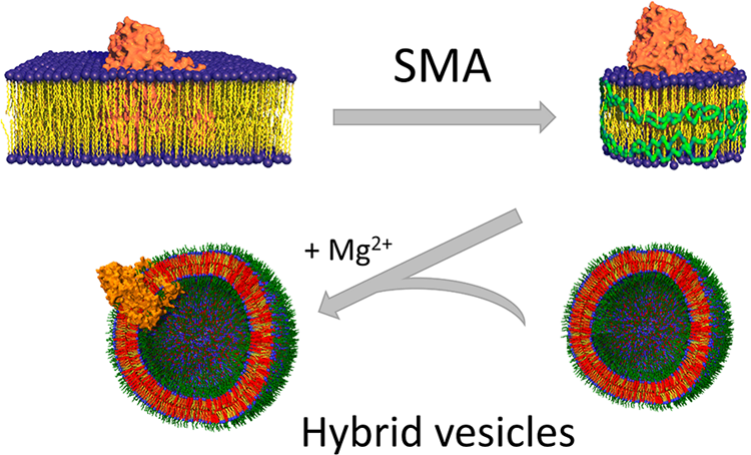

Hybrid vesicles (HVs) that consist of mixtures of block copolymers and lipids are robust biomimetics of liposomes, providing a valuable building block in bionanotechnology, catalysis, and synthetic biology. However, functionalization of HVs with membrane proteins remains laborious and expensive, creating a significant current challenge in the field. Here, using a new approach of extraction with styrene-maleic acid (SMA), we show that a membrane protein (cytochrome *bo*_3_) directly transfers into HVs with an efficiency of 73.9 ± 13.5% without the requirement of detergent, long incubation times, or mechanical disruption. Direct transfer of membrane proteins using this approach was not possible into liposomes, suggesting that HVs are more amenable than liposomes to membrane protein incorporation from a SMA lipid particle system. Finally, we show that this transfer method is not limited to cytochrome *bo*_3_ and can also be performed with complex membrane protein mixtures.

## Introduction

Vesicles made of natural or synthetic lipids (liposomes) are a suitable platform for mimicking membrane structures and functions found in nature.^1,2^ Liposomes have been widely exploited to fabricate artificial compartments in bottom-up synthetic biology (artificial cells and organelles) and nanoreactors in compartmentalized (photo)catalysis.^3,4^ Functionalization of liposomes in biotechnology is achieved by the reconstitution of membrane proteins (MPs), which in spite of their complex amphiphilic nature, have an increasing number of promising applications in areas such as drug discovery,^5^ vaccines,^6^ biosensors,^7^ and energy conversion.^8^ However, the application of proteoliposomes is still hampered by the lack of chemical and physical long-term stability (typically days)^9^ and the complexity of purification and reconstitution of MPs.^10,11^

Recent developments using amphiphilic polymers have shown promise in solving these experimental limitations. Amphiphilic polymers can self-assemble into robust and stable vesicles, known as polymersomes.^12,13^ Despite the advantageous stability and tunability of these synthetic vesicles,^14^ the non-native polymeric environment can limit the functional incorporation of many MPs.^15^ Hybrid vesicles (HVs), composed of a mixture of block copolymers and lipids, have proven to be a balanced compromise between liposome biocompatibility and polymersome stability.^16−20^ Several block copolymers have been studied to correlate how their chemical structure affects the overall properties of the HVs, and both well-mixed and phase-separated membranes have been used.^15,21,22^ We have previously shown that the membrane protein cytochrome *bo*_3_ (cyt *bo*_3_) can be functionally reconstituted into HVs containing up to 50 mol % of the diblock copolymer poly(butadiene-*b*-ethylene oxide) (PBd_22_-*b*-PEO_14_) with POPC lipids, with minimal loss in protein activity and enhanced lifetime up to 500 days.^16,23^

Despite the promise of polymersomes and HVs, the process of extraction, purification, and functional reconstitution of MPs still presents major challenges. Reconstitution methods into polymersomes and HVs are based on methods developed for reconstitution in liposomes, which require detergents and often extensive optimization. Detergents can destabilize MPs by inducing protein unfolding, dissociation of small subunits, and removal of natural lipids associated with the protein hydrophobic regions, and consequently compromise their activity and limit their functional lifetime.^24−26^ Thus, the selection of a compatible detergent and optimum condition to extract a target protein can be a laborious, time-consuming, and risk-prone procedure.^27,28^

**Figure 1:**
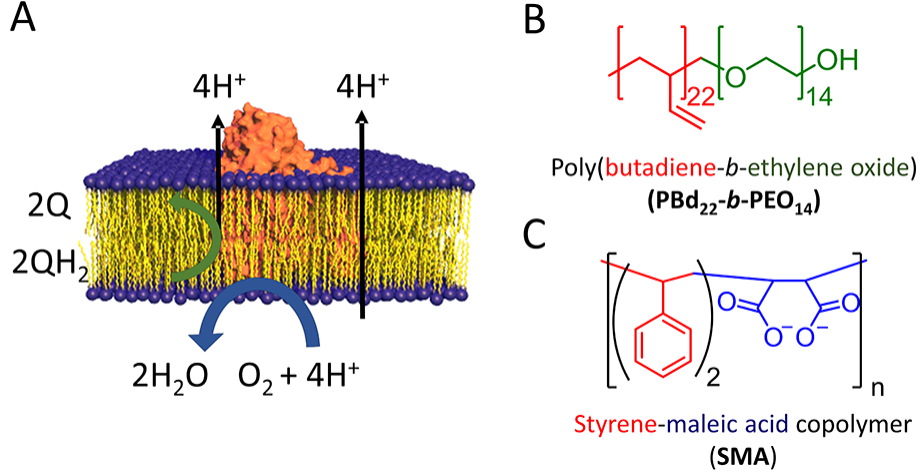
(A) Schematic representation of the structure and function of cyt *bo*_3_ (orange) embedded in the lipid bilayer (represented with yellow lipid tails and blue head groups). (B) Chemical structures of PBd_22_-*b*-PEO_14_ copolymer, with the polybutadiene block polymer in red and the polyethylene glycol block polymer in green. (C) SMA (2:1) copolymer, with the styrene group in red and the maleic acid group in blue.

Here, we report a novel strategy for the reconstitution of a membrane protein, cyt *bo*_3_, from *Escherichia coli* (Figure 1A), into HVs. Cyt *bo*_3_ is a four-subunit membrane enzyme complex (∼143 kDa) from *E. coli* that belongs to the heme-copper oxidase enzyme family and, as such, accepts electrons from ubiquinol and passes them onto molecular oxygen, coupling the electron transfer with proton pumping across the membrane (Figure 1A).^29^ Activity of cyt *bo*_3_, and thus functional reconstitution into the membrane vesicles, is commonly evaluated by measuring oxygen consumption. For the HVs, we selected PBd_22_-*b*-PEO_14_ (MW 1.8 kDa) (Figure 1B), as this copolymer is a compromise between the stability of higher MW polymers and minimizing the difference in hydrophobic thickness between the membranes of pure polymer and pure lipid systems and forms a homogeneous blend with lipids.^15,30^

Using a novel procedure, we show that reconstitution of cyt *bo*_3_ into HVs does not require the use of a detergent. Instead, insertion of cyt *bo*_3_ into the HVs is accomplished by a second amphiphilic polymer, styrene-maleic acid copolymer (SMA, Figure 1C). SMA and similar polymers have emerged as an effective material to extract and solubilize MPs, including cyt *bo*_3_,^31^ while preserving protein activity,^32^ overcoming issues encountered with detergent-mediated solubilization.^33,34^ SMA is an anionic copolymer containing carboxylic acid pendant groups in the form of maleic acid alternating with the hydrophobic styrene pendant groups (Figure 1C).

Unlike detergents, SMA copolymers do not self-assemble into micelles.^35^ When added to cellular membrane extracts, the hydrophobic styrene groups of SMA copolymers intercalate between the acyl chains of the lipid bilayer, whereas the hydrophilic maleic acid groups interface with the solvent.^32^ This interaction between SMA copolymers and membranes leads to the spontaneous formation of discoidal particles of ∼10 nm diameter.^36^ SMA copolymers offer the advantage of solubilizing MPs directly from the cell membrane by forming these nanodisc structures, called SMA–lipid particles (SMALPs), which retain the natural lipids associated with the MPs.^37,38^ MPs can be purified from SMALPs by affinity chromatography.^39^ Besides their use for structural and functional studies,^39^ SMALPs have recently been shown to mediate reconstitution of MPs into planar lipid bilayers, as the tetrameric K^+^ channel,^40^ and into liposomes, as exemplified for a cytochrome *c* oxidase^41^ and a Na^+^/H^+^ antiporter.^42^ In addition to SMA, other maleic acid copolymers capable of solubilizing MPs have been synthesized with various chemical functionalities, such as aliphatic side chains replacing the styrene group^43−45^ or differently charged moieties in the maleic group, providing a diverse toolkit of potential polymers.^45−47^

## Results

First, we investigated the stability of HVs when exposed to increasing concentrations of SMA copolymer (Figures S2 and S3). SMA is seen to solubilize HVs at an SMA to lipid and PBd_22_-*b*-PEO_14_ copolymer ratio of 1 (mol_SMA_/mol_(Lipids+PBd22-*b*-PEO14)_), with less SMA needed to solubilize HVs than liposomes. Still, the amount of SMA required to reconstitute cyt *bo*_3_ is about 20 time less (see below), and thus we excluded that the presence of SMA during the reconstitution of cyt *bo*_3_ could affect the stability of the hybrid vesicles.

SMA-solubilized cyt *bo*_3_ (SMA_cyt *bo*3_) were prepared from membrane extracts of *E. coli* GO105/pJRhisA^48^ (protein content ∼4 mg/mL), containing His-tagged cyt *bo*_3_, by incubation with 2% (w/v) SMA for 2 h at room temperature (RT) and purified via Ni-NTA affinity chromatography (as described in the Supporting Information). Purity of SMA_cyt *bo*3_ was confirmed in a direct comparison with a previous published procedure^48^ using *n*-dodecyl-β-d-maltoside (Figure S1, DDM_cyt *bo*3_).

SMA_cyt *bo*3_ and DDM_cyt *bo*3_ were reconstituted into HVs and lipid-only liposomes (*E. coli* “polar” lipid extract, LIP). As such, four vesicle samples are compared, which hereafter will be named (1) HV-SMA_cyt *bo*3_, (2) HV-DDM_cyt *bo*3_, (3) LIP-SMA_cyt *bo*3_ and (4) LIP-DDM_cyt *bo*3_. HVs were composed of PBd_22_-*b*-PEO_14_ and *E. coli* “polar” lipid extracts at a 1:1 mol/mol ratio.

Reconstitution of DDM_cyt *bo*3_ into HV-DDM_cyt *bo*3_ and LIP-DDM_cyt *bo*3_ was performed by destabilization with detergent (Triton X-100), followed by extensive removal of the detergent by Biobeads, as previously reported^16^ (described in the Supporting Information). To reconstitute SMA_cyt *bo*3_, we took advantage of SMA precipitating in the presence of MgCl_2_ (>5 mM) due to the interactions of the divalent cation Mg^2+^ with the maleic acid groups.^49^ Without the SMA belt, the lipid particles become unstable and will precipitate with the contained MP, unless reconstituted. This strategy has previously been used to exchange the membrane protein AcrB from SMALP into an amphipol scaffold.^38^ SMA_cyt *bo*3_ was incubated with HVs (or liposomes as control) on ice for 30 min at a protein to lipid ratio of ∼1:100 (w/w) and then incubated with 10 mM MgCl_2_ to precipitate SMA. Cyt *bo*_3_ that was not reconstituted into HVs or liposomes was removed by centrifugation at 17000*g* for 15 min. Treatment with 10 mM MgCl_2_ does not affect the size of the vesicles (Figure S4).

**Figure 2:**
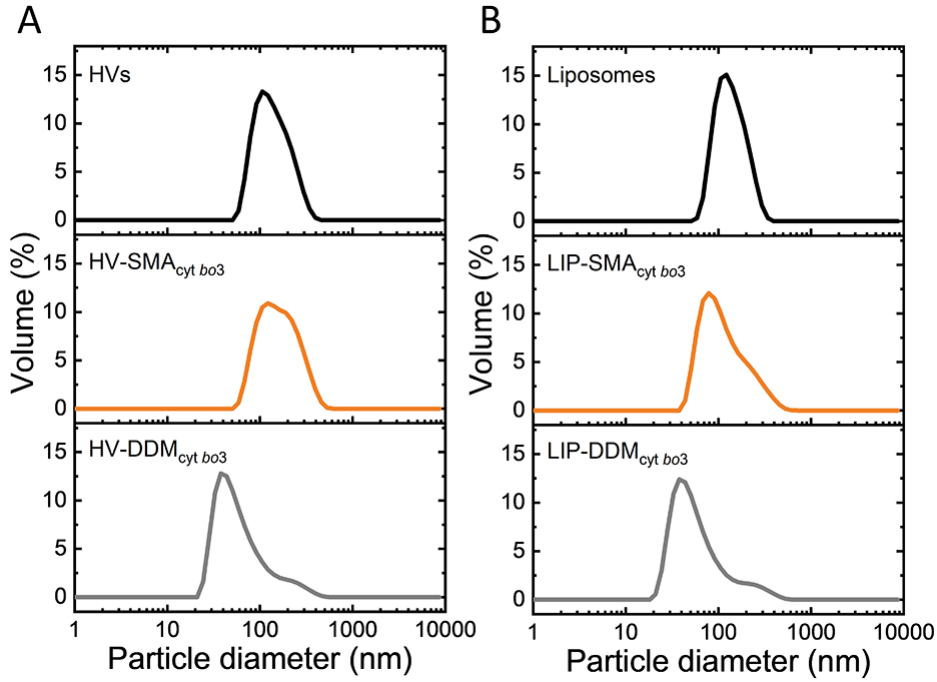
Physical characterization of membrane vesicles. Dynamic light scattering (DLS) volume profiles of (A) HVs, HV-SMA_cyt *bo*3_, and HV-DDM_cyt *bo*3_ and (B) liposomes, LIP-SMA_cyt *bo*3_, and LIP-DDM_cyt *bo*3_. The concentration of analyzed samples was 0.5 mg/mL of total PBd_22_-*b*-PEO_14_ polymer and lipid components.

Dynamic light scattering (DLS) analysis of the four reconstituted samples in Figure 2 (see Table S1 for details) showed that the diameter of the HVs (Figure 2A) slightly increased after SMA_cyt *bo*3_ reconstitution (from ∼130 nm to ∼150 nm), but otherwise remain largely unaltered. In contrast, DDM_cyt *bo*3_ reconstitution into HV shows a clear reduction in liposome size and an increase in polydispersity (see Table S1). The same is observed for the reconstitution of DDM_cyt *bo*3_ in liposomes (Figure 2B). The decreases in size suggest that the Biobead treatment might extract lipids from the HVs and liposomes. The reason for the increase in polydispersity during the DDM reconstitution is unknown, but we hypothesize that some cyt *bo*_3_ might not properly have reconstituted, causing some aggregation in the sample.

**Figure 3:**
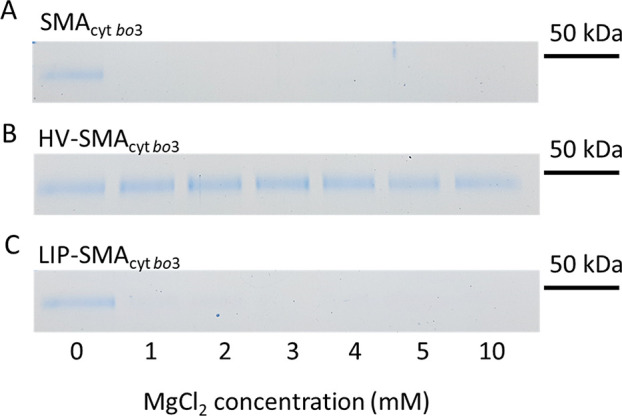
Analysis of (A) SMA_cyt *bo*3_, (B) HV-SMA_cyt *bo*3_, and (C) LIP-SMA_cyt *bo*3_. After direct incubation of SMA_cyt *bo*3_ with HVs or liposomes, samples were incubated with increasing Mg^2+^ concentration for 2 h, followed by centrifugation at 17000*g* for 15 min to pellet nonreconstituted SMA_cyt *bo*3_. The supernatant containing HVs or liposomes was analyzed with SDS-PAGE (Coomassie Blue staining). Only subunit I of cyt *bo*_3_ is shown. The entire gel is shown in Figure S5.

The reconstitution efficiency of cyt *bo*_3_ was quantified by solubilization of the vesicles with Triton X-100 and UV analysis of the Soret peak of cyt *bo*_3_ (409 nm). Interestingly, the reconstitution efficiency of SMA_cyt*bo*__3_ was profoundly different between HVs and liposomes (Table 1). SMA_cyt *bo*3_ could be directly reconstituted into HVs but not into liposomes. This difference in reconstitution efficiency between HVs and liposomes was also confirmed by sodium dodecyl sulfate polyacrylamide gel electrophoresis (SDS-PAGE) (Figure 3 and Figure S5).

**Table 1:** Reconstitution Efficiency of SMA_cyt *bo*__3_ and DDM_cyt *bo*__3_ in Vesicles As Quantified by UV–Vis Spectroscopy of the Soret Band (409 nm)

vesicle sample	reconstitution efficiency (%)	±SD
HV-SMA_cyt *bo*3_	73.9	±13.5
LIP-SMA_cyt *bo*3_	not detected	
HV-DDM_cyt *bo*3_	61.0	±7.5
LIP-DDM_cyt *bo*3_	58.0	±3.5

**Figure 4:**
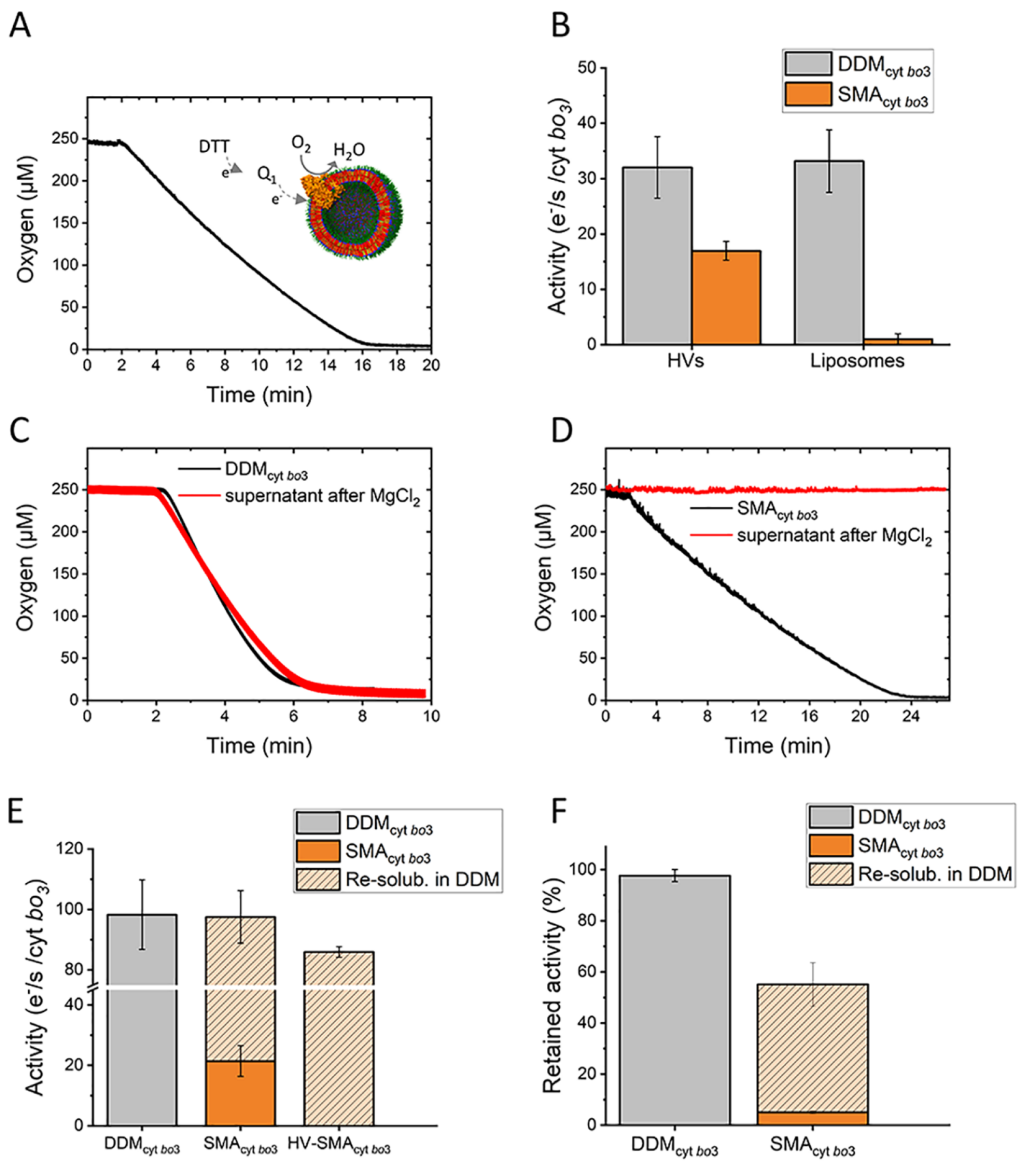
(A) Oxygen consumption trace for HV-SMA_cyt *bo*3_. The oxygen consumption rate was determined via regression of the first 30 s from the slope and normalized by the protein concentration. (B) Comparison of the activities of reconstituted cyt *bo*_3_ determined via oxygen consumption. Error bars represent the standard deviation of three independent experiments. (C) Oxygen consumption traces for DDM_cyt bo3_ and (D) SMA_cyt bo3_. The traces show the activity before and after MgCl_2_ treatment. (E) Comparison of the activities of soluble SMA_cyt bo3_ and DDM_cyt bo3_ determined via oxygen consumption. The graph also shows the activity of soluble SMA_cyt bo3_ and HV-SMA_cyt bo3_ after resolubilization in DDM (1%). Error bars represent the standard deviation of three independent experiments. (F) Activity retention after incubation with 10 mM MgCl_2_ and centrifugation for the supernatant fractions of soluble DDM_cyt bo3_, soluble SMA_cyt bo3_, and soluble SMA_cyt bo3_ in the presence of 1% DDM. The activity retention was determined via comparison of the oxygen consumption rate (determined via regression of the first 30 s from the slope and normalized by the protein concentration) before and after MgCl_2_ treatment and centrifugation.

The activities of reconstituted cyt *bo*_3_ were compared by measuring the rates of oxygen consumption with the substrate ubiquinol 1 (Q_1_) (200 μM), which is reduced by dithiothreitol (DTT) (2 mM) (Figure 4A, see Supporting Information for details). Figure 4B shows the activity of SMA_cyt *bo*3_ after reconstitution into either HVs or liposomes. In correspondence with the results above, LIP-SMA_cyt *bo*3_ did not exhibit any substantial enzyme activity, in line with the fact that SMA_cyt *bo*3_ does not reconstitute into liposomes. In contrast, HV-SMA_cyt *bo*3_ shows clear activity, about half that of the control samples HV-DDM_cyt *bo*3_ and LIP-DDM_cyt *bo*3_ (Figure 4B). We note that, before reconstitution, the activity of the soluble SMA_cyt *bo*3_ is significantly lower than the activity of DDM_cyt *bo*3_ (Figure 4C–E). A reduction in activity has been previously reported for other enzymes in SMALPs.^50,51^ The same reduction in activity is also apparent after DDM_cyt *bo*3_ is reconstituted into liposomes (LIP-DDM_cyt *bo*3_). We speculate that this might be an experimental artifact due to differences in substrate access (Q_1_) to the quinol-binding site of the enzyme in DDM micelles vs the enzyme embedded into lipid membranes or SMALPs. Importantly, after resolubilization in 1% DDM detergent of both soluble SMA_cyt *bo*3_ and HV-SMA_cyt *bo*3_, cyt *bo*_3_ regains an activity similar to DDM_cyt *bo*3_ (Figure 4E and F). This confirms that neither the solubilization of cyt *bo*_3_ into SMALPs nor the reconstitution into HVs irreversibly changes cyt *bo*_3_ and supports our hypothesis that the reduction in activity is due to the enzyme assay which utilizes a non-natural substrate analogue, Q_1_. This is further supported by a structure of cyt *bo*_3_ that was shown not to be affected by solubilization with a slightly different SMA copolymer (3:1).^31^

**Figure 5:**
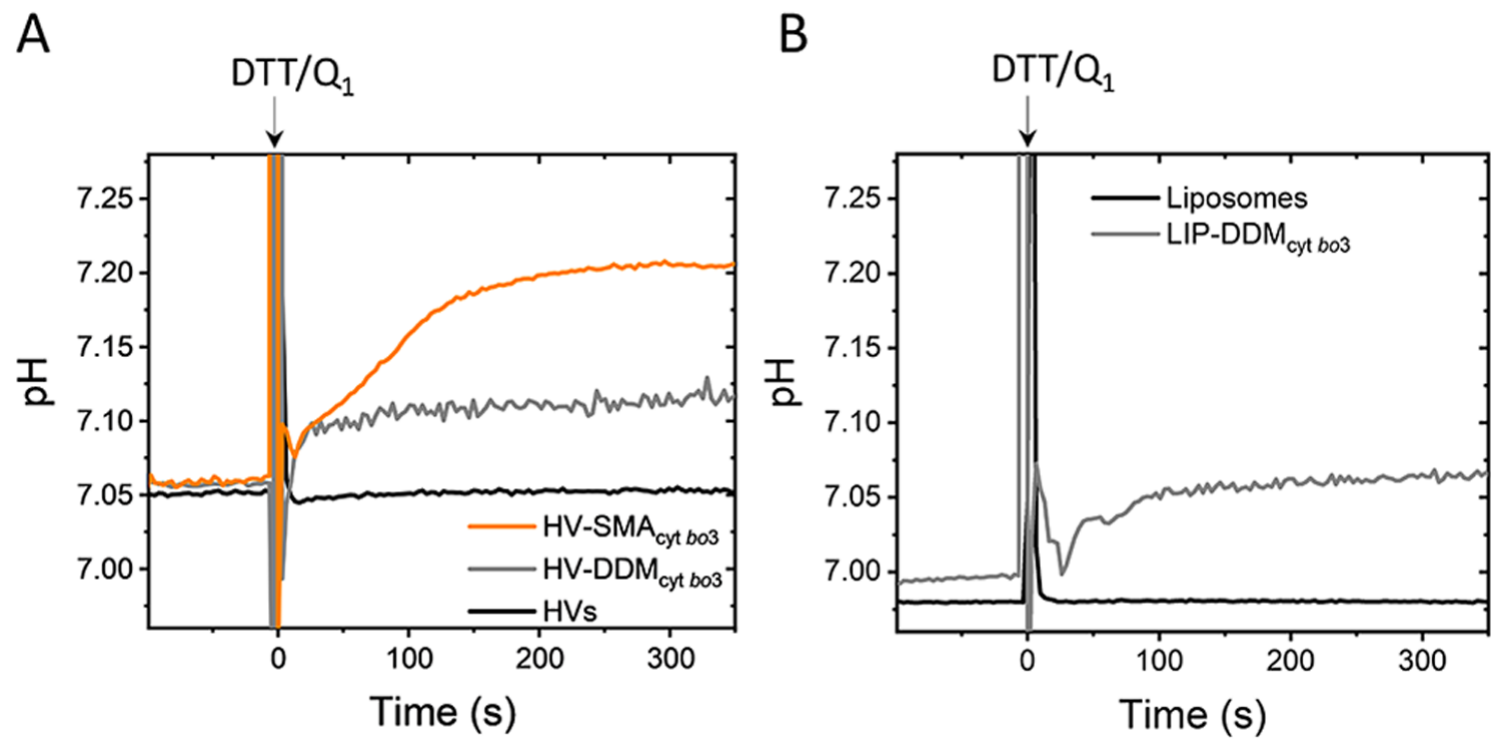
Intravesicular pH change for (A) HVs, HV-SMA_cyt *bo*3_, and HV-DDM_cyt *bo*3_ and (B) liposomes and LIP-DDM_cyt *bo*3_. Displayed curves are representatives of three independent experiments.

In order to confirm that reconstituted cyt *bo*_3_ was fully inserted across the membranes of HVs, we evaluated the net change in intravesicular pH due to the proton-pumping activity of the enzyme upon chemical activation. Changes in internal pH were determined by ratiometric fluorescence measurements of the pH-sensitive fluorescent probe 8-hydroxypyrene-1,3,6-trisulfonic acid (HPTS) (Figure S6, see Supporting Information for details). While HVs showed a constant intravesicular pH after the addition of DTT and Q_1_, both HV-SMA_cyt *bo*3_ and HV-DDM_cyt *bo*3_ displayed an increase of intravesicular pH (Figure 5A), similarly to LIP-DDM_cyt *bo*3_ (Figure 5B). The increase in pH indicates that the cyt *bo*_3_ was successfully inserted into the membrane with a prevalence of an “outward” orientation, as previously demonstrated in liposomal reconstitution.^52,53^

To further assess the ability of SMA to facilitate the reconstitution of membrane proteins (MPs) into HVs, we attempted the reconstitution of the full MPs composition of *E. coli*. To do this, an *E. coli* membrane extract (GO105/pJRhisA) was solubilized with SMA and nonsolubilized material removed by ultracentrifugation (100000*g* for 60 min). This full extract of all SMALPs was incubated with HVs on ice for 30 min, at a 2:8 protein mass to polymer and lipids mass ratio. MPs not reconstituted into HVs were again precipitated by addition of 10 mM MgCl_2_ and removed by centrifugation (17000*g* for 15 min). We compared the protein solubilization efficiencies of soluble and reconstituted MPs by measuring the protein concentration (bicinchoninic acid (BCA) assay, Table 2). Overall, 52.6 (±4.6)% of the *E. coli* MPs were solubilized by SMA. After reconstitution, more than half of this fraction (29.4 (±6.8)%) was successfully reconstituted into HVs.

**Table 2:** Solubilization Efficiency of *E. coli* Membrane Protein Extract *via* SMALPs and subsequent reconstitution efficiency into HVs^a^

	SMALP fraction	solubilization efficiency (%)	±SD
before MgCl_2_ addition	total	52.6	4.6
after MgCl_2_ and centrifugation	supernatant	<1	<1
	pellet	43.5	8.6
before MgCl_2_ addition	HVs	53.1	2.2
after MgCl_2_ and centrifugation	HVs (supernatant)	29.4	6.8
	HVs (pellet)	21.6	5.3

a Solubilization efficiency was determined by BCA assay and expressed as a percentage of total MP content prior to SMA solubilization.

**Figure 6:**
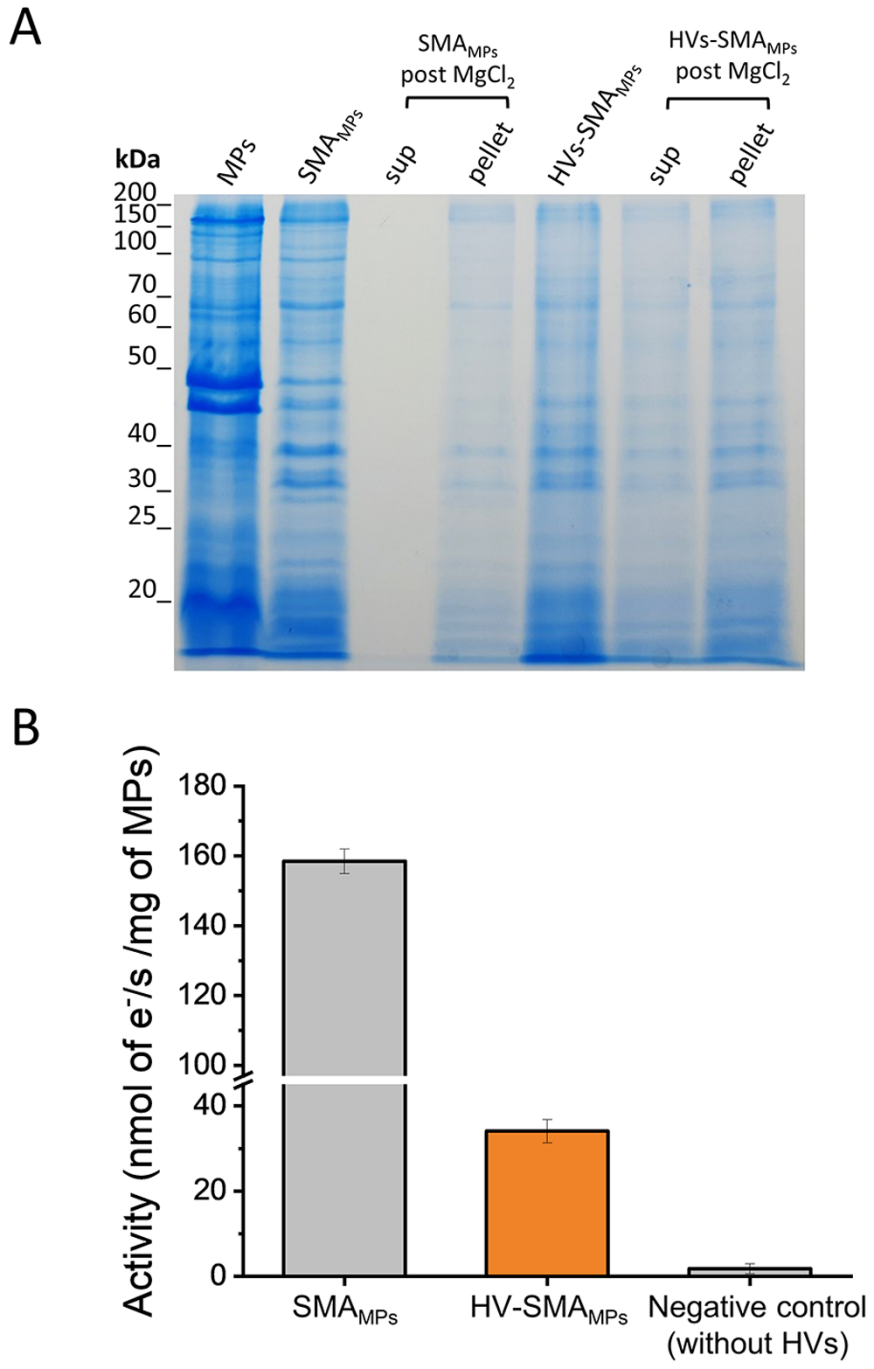
(A) SDS-PAGE (15%) analysis of membrane protein samples contained in either SMA_MPs_ or HV-SMA_MPs_ before and after treatment with MgCl_2_ and separation of the insoluble part via precipitation. The concentration of *E. coli* membrane-protein fraction (MPs, lane 1) was halved in comparison to the other loaded samples to improve the quality of the SDS-PAGE. (B) Comparison of the oxygen-reducing activities of soluble SMA_MPs_, HV-SMA_MPs_, and SMA _MPs_ treated with MgCl_2_ without HVs (“negative control”). The activity is normalized per mg of total MP content for SMA_MPs_ and HV-SMA_MPs_ determined via BCA assay. Error bars represent the standard deviation of three independent experiments.

To assess whether the protein content after reconstitution into HVs was a true representation of the various MPs from native membranes of *E. coli*, we conducted an SDS-PAGE analysis for qualitative comparison (Figure 6A). SDS-PAGE showed very similar profiles for each condition, strongly suggesting that SMA can extract a wide range of membrane proteins and transfer these to HVs. This analysis also confirmed that precipitation of SMALPs with 10 mM MgCl_2_ (i.e., without HVs) removed the entire protein content if not reconstituted. Finally, we evaluated whether the MPs were functionally active after reconstituted into HVs by monitoring the activity of the cyt *bo*_3_, which was part of the MP extract mixture. Figure 6B and Figure S7 show the oxygen reduction activity of the full MP extracts solubilized by SMA before (SMA_MPs_) and after (HV-SMA_MPs_) reconstitution into HVs. The activity confirms that cyt *bo*_3_ was functionally active after transfer into HVs, indicating that complex mixtures of proteins can be reconstituted with SMA. The oxygen reduction activity, normalized against total MP content, is lower after reconstitution in HVs, and we hypothesize that this is due to different efficiencies of reconstitution of the various MPs.

## Discussion and Conclusions

Although SMA-solubilized proteins have previously been shown to reconstitute into planar lipid bilayers^40^ or liposomes,^41,42^ the mechanisms by which this happens is not fully understood. Indeed, little is known about the interaction between SMALPs and lipid membranes, although it has been shown that the lipid packing properties and electrostatic interactions strongly influence how SMA interplays with the lipid bilayer.^54^ Particularly, phospholipid phosphoethanolamine (PE), characterized by a negative intrinsic curvature,^55^ exerts a lateral pressure that hampers SMA insertion and, therefore, membrane solubilization.^54,56,57^ Similarly, we hypothesize that PE might hamper SMA reconstitution of MPs back into liposomes. This may explain the lack of reconstitution of SMA_cyt *bo*3_ into the liposomes in this study, which were prepared from an *E. coli* “polar” lipid extract (PE, ∼65 mol %; PG, ∼25 mol %; and cardiolipin, ∼10 mol %).^15^

We have previously observed that hybrid giant unilamellar vesicles (GUVs) of PBd_22_-*b*-PEO_14_ and 1-palmitoyl-2-oleoyl-sn-glycero-3-phosphocholine (POPC) are well-mixed and homogeneous with a similar molecular ordering and packing, but lower fluidity, than POPC lipid bilayers.^58^ Previous works have also shown that the area stretching moduli (*K*_a_) of polymersomes made of PBd-*b*-PEO polymers (90–130 mN/m^59,60^) are much lower than the typical *K*_a_ for phosphocholine liposomes (200–260 mN/m).^60,61^ For HVs composed of PBd_22_-*b*-PEO_14_ and 1,2-Dioleoyl-sn-glycero-3-phosphocholine (DOPC), or PBd_46_-*b*-PEO_30_ mixed with POPC, the area stretching modulus lies intermediate between that of pure polymer and pure lipid vesicles.^18,60^ While comparable data are not available for mixtures of *E. coli* polar lipid extract and PBd_22_-*b*-PEO_14_, we infer that the block copolymer will impart a similar reduction in the stretching modulus of vesicles in this work. Importantly, the area stretching modulus is proportional to the surface tension (γ) of the membrane (*K*_a_ ∼4γ). The decreased surface tension and reduced work required to stretch the interface likely reduce the energy barrier for the transfer of cyt *bo*_3_ from the SMALPs to the HV membrane. It has previously been hypothesized that this enhanced elasticity of hybrid PBd_22_-*b*-PEO_14_ membranes lowers the energy cost for membrane deformations required to accommodate insertion of the membrane protein.^18^ Thus, here, we consider the higher elasticity of the HV compared to liposomes to be essential for reconstitution of MPs from SMALPs.

In conclusion, we show for the first time the reconstitution of SMA-solubilized membrane protein either as pure isolated protein (SMA_cyt *bo*3_) or as a complex MP mixture (SMA_MPs_), into vesicles without the use of detergents while maintaining protein activity. For cytochrome *c* oxidase, sonication or extrusion was required to induce its reconstitution into liposomes,^41^ while for plasma membrane Na^+^/H^+^ antiporter, a much longer incubation time (overnight) with liposomes of larger diameter (400 nm) was needed and only ∼10% reconstitution was achieved.^42^ In contrast, a simple incubation for 30 min on ice is sufficient to reconstitute SMA_cyt *bo*3_ into HVs, while the same procedure does not lead to a transfer of cyt *bo*_3_ to liposomes. This method provides a new tool to reduce time and cost for enzyme reconstitution processes by avoiding detergent-mediated reconstitution and represents a solid foundation for further development as an enabling technology for MPs in nanomedicine, biocatalysis, and bottom-up synthetic biology.

## Supporting Information Available

Experimental procedures, SDS-PAGE analyses, destabilization profiles, and additional oxygen consumption traces (PDF)

## References

[ref1] Cintas P. Chasing Synthetic Life: A Tale of Forms, Chemical Fossils, and Biomorphs. Angew. Chem. Int. Ed. 2020, 132, 7364–7372. 10.1002/ange.201915853. 32049403

[ref2] Elani Y. Interfacing Living and Synthetic Cells as an Emerging Frontier in Synthetic Biology. Angew. Chem. Int. Ed. 2021, 60 (11), 5602–5611. 10.1002/anie.202006941. PMC7983915 32909663

[ref3] Wang G.; Castiglione K. Light-Driven Biocatalysis in Liposomes and Polymersomes: Where Are We Now ?. Catalysts 2019, 9 (12), 1–25. 10.3390/catal9010012.

[ref4] Pannwitz A.; Klein D. M.; Rodrıguez-Jimenez S.; Casadevall C.; Song H.; Reisner E.; Hammarstro L.; Bonnet S. Roadmap towards Solar Fuel Synthesis at the Water Interface of Liposome Membranes. Chem. Soc. Rev. 2021, 50, 4833–4855. 10.1039/D0CS00737D. 33659967

[ref5] Yin H.; Flynn A. D. Drugging Membrane Protein Interactions Hang. Annu. Rev. Biomed. Eng. 2016, 18, 51–76. 10.1146/annurev-bioeng-092115-025322. 26863923 PMC4968933

[ref6] van der Pol L.; Stork M.; van der Ley P. Outer Membrane Vesicles as Platform Vaccine Technology. Biotechnol. J. 2015, 10 (11), 1689–1706. 10.1002/biot.201400395. 26912077 PMC4768646

[ref7] Alvarez-Malmagro J.; García-Molina G.; De Lacey A. L. Electrochemical Biosensors Based on Membrane-Bound Enzymes in Biomimetic Configurations. Sensors 2020, 20 (12), 1–17. 10.3390/s20123393. PMC7349357 32560121

[ref8] Zhang H.; Catania R.; Jeuken L. J. C. Membrane Protein Modified Electrodes in Bioelectrocatalysis. Catalysts 2020, 10 (12), 1–29. 10.3390/catal10121427.

[ref9] Rideau E.; Dimova R.; Schwille P.; Wurm F. R.; Landfester K. Liposomes and Polymersomes: A Comparative Review towards Cell Mimicking. Chem. Soc. Rev. 2018, 47 (23), 8572–8610. 10.1039/C8CS00162F. 30177983

[ref10] Skrzypek R.; Iqbal S.; Callaghan R. Methods of Reconstitution to Investigate Membrane Protein Function. Methods 2018, 147, 126–141. 10.1016/j.ymeth.2018.02.012. 29454861

[ref11] Amati A. M.; Graf S.; Deutschmann S.; Dolder N.; Von Ballmoos C. Current Problems and Future Avenues in Proteoliposome Research. Biochem. Soc. Trans. 2020, 48 (4), 1473–1492. 10.1042/BST20190966. 32830854

[ref12] Discher D. E.; Eisenberg A. Polymer Vesicles. Science 2002, 967 (5583), 967–973. 10.1126/science.1074972. 12169723

[ref13] Palivan C. G.; Goers R.; Najer A.; Zhang X.; Car A.; Meier W. Bioinspired Polymer Vesicles and Membranes for Biological and Medical Applications. Chem. Soc. Rev. 2016, 45 (2), 377–411. 10.1039/C5CS00569H. 26563574

[ref14] LoPresti C.; Lomas H.; Massignani M.; Smart T.; Battaglia G. Polymersomes: Nature Inspired Nanometer Sized Compartments. J. Mater. Chem. 2009, 19 (22), 3576–3590. 10.1039/b818869f.

[ref15] Beales P. A.; Khan S.; Muench S. P.; Jeuken L. J. C. Durable Vesicles for Reconstitution of Membrane Proteins in Biotechnology. Biochem. Soc. Trans. 2017, 45 (1), 15–26. 10.1042/BST20160019. 28202656 PMC5310719

[ref16] Khan S.; Li M.; Muench S. P.; Jeuken L. J. C.; Beales P. A. Durable Proteo-Hybrid Vesicles for the Extended Functional Lifetime of Membrane Proteins in Bionanotechnology. Chem. Commun. 2016, 52 (73), 11020–11023. 10.1039/C6CC04207D. PMC5048396 27540604

[ref17] Paxton W. F.; McAninch P. T.; Achyuthan K. E.; Shin S. H. R.; Monteith H. L. Monitoring and Modulating Ion Traffic in Hybrid Lipid/Polymer Vesicles. Colloids Surf., B 2017, 159, 268–276. 10.1016/j.colsurfb.2017.07.091. 28800466

[ref18] Jacobs M. L.; Boyd M. A.; Kamat N. P. Diblock Copolymers Enhance Folding of a Mechanosensitive Membrane Protein during Cell-Free Expression. Proc. Natl. Acad. Sci. U. S. A. 2019, 116 (10), 4031–4036. 10.1073/pnas.1814775116. 30760590 PMC6410776

[ref19] Marušič N.; Otrin L.; Zhao Z.; Lira R. B.; Kyrilis F. L.; Hamdi F.; Kastritis P. L.; Vidaković-Koch T.; Ivanov I.; Sundmacher K.; Dimova R. Constructing Artificial Respiratory Chain in Polymer Compartments: Insights into the Interplay between Bo3 Oxidase and the Membrane. Proc. Natl. Acad. Sci. U. S. A. 2020, 117 (26), 15006–15017. 10.1073/pnas.1919306117. 32554497 PMC7334566

[ref20] Rottet S.; Iqbal S.; Beales P. A.; Lin A.; Lee J.; Rug M.; Scott C.; Callaghan R. Characterisation of Hybrid Polymersome Vesicles Containing the Efflux Pumps NaAtm1 or P-Glycoprotein. Polymers 2020, 12 (5), 1049 10.3390/polym12051049. PMC7284524 32375237

[ref21] Schulz M.; Binder W. H. Mixed Hybrid Lipid/Polymer Vesicles as a Novel Membrane Platform. Macromol. Rapid Commun. 2015, 23 (36), 2031–2041. 10.1002/marc.201500344. 26457675

[ref22] Le Meins J. F.; Schatz C.; Lecommandoux S.; Sandre O. Hybrid Polymer/Lipid Vesicles: State of the Art and Future Perspectives. Mater. Today 2013, 16 (10), 397–402. 10.1016/j.mattod.2013.09.002.

[ref23] Seneviratne R.; Khan S.; Moscrop E.; Rappolt M.; Muench S. P.; Jeuken L. J. C.; Beales P. A. A Reconstitution Method for Integral Membrane Proteins in Hybrid Lipid-Polymer Vesicles for Enhanced Functional Durability. Methods 2018, 147, 142–149. 10.1016/j.ymeth.2018.01.021. 29410153

[ref24] Moraes I.; Evans G.; Sanchez-Weatherby J.; Newstead S.; Shaw Stewart P. D. Membrane Protein Structure Determination — The next Generation. Biochim. Biophys. Acta - Biomembranes 2014, 1838 (1), 78–87. 10.1016/j.bbamem.2013.07.010. PMC3898769 23860256

[ref25] Seddon A. M.; Curnow P.; Booth P. J. Membrane Proteins, Lipids and Detergents : Not Just a Soap Opera. Biochim. Biophys. Acta 2004, 1666 (1–2), 105–117. 10.1016/j.bbamem.2004.04.011. 15519311

[ref26] Palsdottir H.; Hunte C. Lipids in Membrane Protein Structures. Biochim. Biophys. Acta 2004, 1666 (1–2), 2–18. 10.1016/j.bbamem.2004.06.012. 15519305

[ref27] Privé G. G. Detergents for the Stabilization and Crystallization of Membrane Proteins. Methods 2007, 41 (4), 388–397. 10.1016/j.ymeth.2007.01.007. 17367711

[ref28] Postis V. L. G.; Deacon S. E.; Roach P. C. J.; Wright G. S. A.; Xia X.; Wright G. S. A.; Xia X.; Ingram J. C.; Hadden J. M.; Peter J.; Henderson F.; Phillips S. E. V; Roach P. C. J.; Mcpherson M. J.; Baldwin S. A. A High-Throughput Assay of Membrane Protein Stability. Mol. Membr. Biol. 2008, 25 (8), 617–624. 10.1080/09687680802530469. 19016381

[ref29] Garcia-Horsman A. J.; Barquera B.; Rumbley J.; Ma J.; Gennis R. B. The Superfamily of Heme-Copper Respiratory Oxidases. J. Bacteriol. 1994, 176 (18), 5587–5600. 10.1128/jb.176.18.5587-5600.1994. 8083153 PMC196760

[ref30] Lim S. K.; de Hoog H.-P.; Parikh A. N.; Nallani M.; Liedberg B. Hybrid, Nanoscale Phospholipid/Block Copolymer Vesicles. Polymers 2013, 5 (3), 1102–1114. 10.3390/polym5031102.

[ref31] Li J.; Han L.; Vallese F.; Ding Z.; Choi S. K.; Hong S.; Luo Y.; Liu B.; Chan C. K.; Tajkhorshid E.; Zhu J.; Clarke O.; Zhang K.; Gennis R. Cryo-EM Structures of Escherichia Coli Cytochrome Bo 3 Reveal Bound Phospholipids and Ubiquinone-8 in a Dynamic Substrate Binding Site. Proc. Natl. Acad. Sci. U. S. A. 2021, 118 (34), e2106750118 10.1073/pnas.2106750118. 34417297 PMC8403832

[ref32] Lee S. C.; Knowles T. J.; Postis V. L. G.; Jamshad M.; Parslow R. A.; Lin Y.; Goldman A.; Sridhar P.; Overduin M.; Muench S. P.; Dafforn T. R. A Method for Detergent-Free Isolation of Membrane Proteins in Their Local Lipid Environment. Nat. Protoc. 2016, 11 (7), 1149–1162. 10.1038/nprot.2016.070. 27254461

[ref33] Knowles T. J.; Finka R.; Smith C.; Lin Y.; Dafforn T.; Overduin M. Membrane Proteins Solubilized Intact in Lipid Containing Nanoparticles Bounded by Styrene Maleic Acid Copolymer. J. Am. Chem. Soc. 2009, 131 (22), 7484–7485. 10.1021/ja810046q. 19449872

[ref34] Jamshad M.; Charlton J.; Lin Y.; Routledge S. J.; Bawa Z.; Knowles T. J.; Overduin M.; Dekker N.; Dafforn T. R.; Bill R. M.; Poyner D. R.; Wheatley M. G-Protein Coupled Receptor Solubilization and Purification for Biophysical Analysis and Functional Studies, in the Total Absence of Detergent. Biosci. Rep. 2015, 35 (2), e00188 10.1042/BSR20140171. 25720391 PMC4400634

[ref35] Tonge S. R.; Tighe B. J. Responsive Hydrophobically Associating Polymers : A Review of Structure and Properties. Adv. Drug Delivery Rev. 2001, 53 (1), 109–122. 10.1016/S0169-409X(01)00223-X. 11733120

[ref36] Esmaili M.; Overduin M. Membrane Biology Visualized in Nanometer-Sized Discs Formed by Styrene Maleic Acid Polymers. Biochim. Biophys. Acta - Biomembranes 2018, 1860 (2), 257–263. 10.1016/j.bbamem.2017.10.019. 29056560 PMC5908709

[ref37] Orekhov P. S.; Bozdaganyan M. E.; Voskoboynikova N.; Mulkidjanian A. Y.; Steinhoff H.-J.; Shaitan K. V. Styrene/Maleic Acid Copolymers Form SMALPs by Pulling Lipid Patches out of the Lipid Bilayer. Langmuir 2019, 35 (10), 3748–3758. 10.1021/acs.langmuir.8b03978. 30773011

[ref38] Hesketh S. J.; Klebl D. P.; Higgins A. J.; Thomsen M.; Pickles I. B.; Sobott F.; Sivaprasadarao A.; Postis V. L. G.; Muench S. P. Styrene Maleic-Acid Lipid Particles (SMALPs) into Detergent or Amphipols: An Exchange Protocol for Membrane Protein Characterisation. Biochim. Biophys. Acta - Biomembranes 2020, 1862 (5), 183192 10.1016/j.bbamem.2020.183192. 31945320 PMC7086155

[ref39] Pollock N. L.; Lee S. C.; Patel J. H.; Gulamhussein A. A.; Rothnie A. J. Structure and Function of Membrane Proteins Encapsulated in a Polymer-Bound Lipid Bilayer. Biochim. Biophys. Acta - Biomembranes 2018, 1860 (4), 809–817. 10.1016/j.bbamem.2017.08.012. 28865797

[ref40] Dörr J. M.; Koorengevel M. C.; Schäfer M.; Prokofyev A. V.; Scheidelaar S.; van der Cruijesn E. A. W.; Dafforn T. R.; Baldus M.; Killian A. J. Detergent-Free Isolation, Characterization, and Functional Reconstitution of a Tetrameric K+ Channel: The Power of Native Nanodiscs. Proc. Natl. Acad. Sci. U. S. A. 2014, 111 (52), 18607–18612. 10.1073/pnas.1416205112. 25512535 PMC4284610

[ref41] Smirnova I. A.; Ädelroth P.; Brzezinski P. Extraction and Liposome Reconstitution of Membrane Proteins with Their Native Lipids without the Use of Detergents. Sci. Rep. 2018, 8 (1), 14950 10.1038/s41598-018-33208-1. 30297885 PMC6175888

[ref42] Dutta D.; Esmaili M.; Overduin M.; Fliegel L. Expression and Detergent Free Purification and Reconstitution of the Plant Plasma Membrane Na+/H+ Antiporter SOS1 Overexpressed in Pichia Pastoris. Biochim. Biophys. Acta - Biomembranes 2020, 1862 (3), 183111 10.1016/j.bbamem.2019.183111. 31678368

[ref43] Oluwole A. O.; Danielczak B.; Meister A.; Babalola J. O.; Vargas C.; Keller S. Solubilization of Membrane Proteins into Functional Lipid-Bilayer Nanodiscs Using a Diisobutylene/Maleic Acid Copolymer. Angew. Chem. Int. Ed. 2017, 56 (7), 1919–1924. 10.1002/anie.201610778. PMC5299484 28079955

[ref44] Gulamhussein A. A.; Uddin R.; Tighe B. J.; Poyner D. R.; Rothnie A. J. A Comparison of SMA (Styrene Maleic Acid) and DIBMA (Di-Isobutylene Maleic Acid) for Membrane Protein Purification. Biochim. Biophys. Acta - Biomembranes 2020, 1862 (7), 183281 10.1016/j.bbamem.2020.183281. 32209303

[ref45] Barniol-Xicota M.; Verhelst S. H. L. Lipidomic and In-Gel Analysis of Maleic Acid Co-Polymer Nanodiscs Reveals Differences in Composition of Solubilized Membranes. Commun. Biol. 2021, 4 (1), 218 10.1038/s42003-021-01711-3. 33594255 PMC7886889

[ref46] Di Mauro G. M.; La Rosa C.; Condorelli M.; Ramamoorthy A. Benchmarks of SMA-Copolymer Derivatives and Nanodisc Integrity. Langmuir 2021, 37 (10), 3113–3121. 10.1021/acs.langmuir.0c03554. 33645999

[ref47] Hall S. C. L.; Tognoloni C.; Charlton J.; Bragginton É. C.; Rothnie A. J.; Sridhar P.; Wheatley M.; Knowles T. J.; Arnold T.; Edler K. J.; Dafforn T. R. An Acid-Compatible Co-Polymer for the Solubilization of Membranes and Proteins into Lipid Bilayer-Containing Nanoparticles. Nanoscale 2018, 10 (22), 10609–10619. 10.1039/C8NR01322E. 29845165 PMC5996351

[ref48] Rumbley J. N.; Nickels E. F.; Gennis R. B. One-Step Purification of Histidine-Tagged Cytochrome Bo3 from Escherichia Coli and Demonstration That Associated Quinone Is Not Required for the Structural Integrity of the Oxidase. Biochim. Biophys. Acta - Protein Struct. Mol. Enzym. 1997, 1340 (1), 131–142. 10.1016/S0167-4838(97)00036-8. 9217023

[ref49] Morrison K. A.; Akram A.; Mathews A.; Khan Z. A.; Patel J. H.; Zhou C.; Hardy D. J.; Moore-kelly C.; Patel R.; Odiba V.; Knowles T. J.; Javed M.-H.; Chmel N. P.; Dafforn T. R.; Rothnie A. J. Membrane Protein Extraction and Purification Using Styrene – Maleic Acid (SMA) Copolymer: Effect of Variations in Polymer Structure. Biochem. J. 2016, 473 (23), 4349–4360. 10.1042/BCJ20160723. 27694389

[ref50] Liu Y.; Moura E. C. C. M.; Dörr J. M.; Scheidelaar S.; Heger H.; Egmond M. R.; Killian J. A.; Mohammadi T.; Breukink E. Bacillus Subtilis MraY in Detergent-Free System of Nanodiscs Wrapped by Styrene-Maleic Acid Copolymers. PLoS One 2018, 13 (11), 1–18. 10.1371/journal.pone.0206692. PMC6218056 30395652

[ref51] Barniol-Xicota M.; Verhelst S. H. L. Stable and Functional Rhomboid Proteases in Lipid Nanodiscs by Using Diisobutylene/Maleic Acid Copolymers. J. Am. Chem. Soc. 2018, 140 (44), 14557–14561. 10.1021/jacs.8b08441. 30347979

[ref52] Li M.; Jørgensen S. K.; Mcmillan D. G. G.; Krzemiński Ł. K.; Daskalakis N. N.; Partanen R. H.; Tutkus M.; Tuma R.; Stamou D.; Hatzakis N. S.; Jeuken L. J. C. Single Enzyme Experiments Reveal a Long-Lifetime Proton Leak State in a Heme-Copper Oxidase. J. Am. Chem. Soc. 2015, 137 (51), 16055–16063. 10.1021/jacs.5b08798. 26618221 PMC4697922

[ref53] Mazurenko I.; Hatzakis N. S.; Jeuken L. J. C. Single Liposome Measurements for the Study of Proton-Pumping Membrane Enzymes Using Electrochemistry and Fluorescent Microscopy. J. Visualized Exp. 2019, (144), 1–11. 10.3791/58896. 30855567

[ref54] Scheidelaar S.; Koorengevel M. C.; Pardo J. D.; Meeldijk J. D.; Breukink E.; Killian J. A. Molecular Model for the Solubilization of Membranes into Nanodisks by Styrene Maleic Acid Copolymers. Biophys. J. 2015, 108 (2), 279–290. 10.1016/j.bpj.2014.11.3464. 25606677 PMC4302193

[ref55] Kollmitzer B.; Heftberger P.; Rappolt M.; Pabst G. Monolayer Spontaneous Curvature of Raft-Forming Membrane Lipids. Soft Matter 2013, 9 (45), 10877–10884. 10.1039/c3sm51829a. 24672578 PMC3963256

[ref56] Dörr J. M.; Scheidelaar S.; Koorengevel M. C.; Dominguez J. J.; Schäfer M.; van Walree C. A.; Killian J. A. The Styrene–Maleic Acid Copolymer: A Versatile Tool in Membrane Research. Eur. Biophys. J. 2016, 45 (1), 3–21. 10.1007/s00249-015-1093-y. 26639665 PMC4698303

[ref57] Dominguez Pardo J. J.; Dörr J. M.; Iyer A.; Cox R. C.; Scheidelaar S.; Koorengevel M. C.; Subramaniam V.; Killian J. A. Solubilization of Lipids and Lipid Phases by the Styrene–Maleic Acid Copolymer. Eur. Biophys. J. 2017, 46 (1), 91–101. 10.1007/s00249-016-1181-7. 27815573 PMC5209432

[ref58] Seneviratne R.; Catania R.; Rappolt M.; Jeuken L. J. C.; Beales P. A. Membrane Mixing and Dynamics in Hybrid POPC/Poly(1,2-Butadiene- *Block* -Ethylene Oxide) (PBd-*b* -PEO) Lipid/Block Co-Polymer Giant Vesicles. Soft Matter 2022, 18, 1294 10.1039/D1SM01591E. 35048939

[ref59] Bermudez H.; Brannan A. K.; Hammer D. A.; Bates F. S.; Discher D. E. Molecular Weight Dependence of Polymersome Membrane Structure, Elasticity, and Stability. Macromolecules 2002, 35 (21), 8203–8208. 10.1021/ma020669l.

[ref60] Nam J.; Beales P. A.; Vanderlick T. K. Giant Phospholipid/Block Copolymer Hybrid Vesicles: Mixing Behavior and Domain Formation. Langmuir 2011, 27 (1), 1–6. 10.1021/la103428g. 21133340

[ref61] Rawicz W.; Olbrich K. C.; Mcintosh T.; Needham D.; Evans E. Effect of Chain Length and Unsaturation on Elasticity of Lipid Bilayers. Biophys. J. 2000, 79, 328–339. 10.1016/S0006-3495(00)76295-3. 10866959 PMC1300937

