# Supplementary material for: Detergent-Free Functionalization of Hybrid Vesicles with Membrane Proteins Using SMALPs
Source: Macromolecules. 2022 Apr 21;55(9):3415–22. doi: 10.1021/acs.macromol.2c00326 (PMC9097535; doi:10.1021/acs.macromol.2c00326)
Supplement: Supplementary file 1 — ma2c00326_si_001.pdf [file ma2c00326_si_001.pdf]

Supporting Information

Detergent-free functionalization of hybrid vesicles with membrane proteins using SMALPs

Rosa Catania,[†,‡] Jonathan Machin,[†,‡] Michael Rappolt,[§] Stephen P. Muench,[†,‡] Paul A. Beales,*[†,^] and Lars J.C. Jeuken*[†,‡,‖]

[†] Astbury Centre of Structural Molecular Biology, University of Leeds, Leeds LS2 9JT (UK)

[‡] School of Biomedical Sciences, University of Leeds, Leeds LS2 9JT (UK)

[§] School of Food Science and Nutrition, University of Leeds, Leeds LS2 9JT (UK)

[^] School of Chemistry, University of Leeds, Leeds LS2 9JT (UK)

‖ Leiden Institute of Chemistry, University Leiden, Leiden 2300RA (NL)

* Corresponding authors: P.A.Beales@leeds.ac.uk, L.J.C.Jeuken@lic.leidenuniv.nl

Methods:

Cytochrome *bo₃* extraction and purification. Membrane protein cytochrome bo_3 (cyt bo_3) was expressed in *Escherichia coli* GO105/pJRhisA as previously described.[1] A colony of fresh plated *E. coli* GO105/pJRhisA was inoculated in LB (Lysogeny Broth) medium supplemented with 100 µg/mL carbenicillin and cultured at 37 °C at an agitation rate of 200 rpm for ~16 h. This starter culture was then inoculated in LB medium (2% v/v) supplemented with 100 µg/mL carbenicillin and 0.1 mM $CuSO_4$. *E. coli* was grown to mid-logarithmic phase at 37 °C at an agitation rate of 200 rpm for ~6 h (approximately until optical density of a sample measured at a wavelength of 600 nm, OD_{600nm}, reached 1.5). *E. coli* cells were harvested by centrifugation at 7,000 *g* for 20 min at 4 °C and resuspended in W1 buffer (20 mM MOPS, 5 mM Mg_2SO_4, 30 mM Na_2SO_4) at a concentration of 0.25 g of wet cells per mL. *E. coli* cells suspension was passed twice through a cell disrupter (Constant Systems) at 30 kPsi. Cell debris was removed by centrifugation at 17,500 *g* for 10 min at 4 °C. Cell membranes in the supernatant were spun down by ultracentrifugation at 200,000 *g* for 90 min at 4 °C.

For purification of cyt bo_3 in SMA nanodiscs, the membrane pellet was resuspended in 50 mM Tris-HCl (pH 8), 500 mM NaCl and 10% glycerol at a 'wet weight' concentration of 40

mg/mL (protein content ~ 4 mg/mL). Styrene maleic acid (SMA) copolymer (Cray Valley, SMA 2000 – MW 7.5 kDa) was added at a concentration of 2% w/v; the suspension was incubated for 2 h on a rotary shaker at RT and then centrifuged at 100,000 *g* for 45 min at 4 °C to remove any non-solubilized proteins. The supernatant was used directly for complex membrane proteins mixture (SMA_{MPs}) reconstitution or further purified for isolation of $SMA_{cyt\ bo3}$.

For purification of cyt *bo₃ via* DDM, the membrane pellet was resuspended in 20 mM Tris-Cl (pH 8), 5 mM $MgSO_4$, 300 mM NaCl, 20 mM imidazole, 10% glycerol and 1% DDM at a protein concentration of 4 mg/mL. The suspension was incubated for 2 h on a rotary shaker at 4 °C and then centrifuged at 200,000 *g* for 45 min at 4 °C.

Both SMA-solubilized proteins and DDM-solubilized proteins were incubated with pre-equilibrated Ni^{2+}-NTA resin (Neo Biotech) for ~16 h and 1 h, respectively, on a rotary shaker at 4 °C. The resin suspensions were loaded onto a gravity column, and either the $SMA_{cyt\ bo3}$ nanodiscs or the $DDM_{cyt\ bo3}$ were eluted with 200 mM imidazole (supplemented with 500 mM or 100 mM NaCl, respectively), which was removed immediately after the elution by 3 cycles of dilution in imidazole-free storage buffer and concentration using 100 kDa MW cut-off concentrator (VivaSpin), and stored at -20 °C until use.

The protein concentration of the cytoplasmic membrane preparation was determined using a bicinchoninic acid assay (BCA) assay. Protein concentration of purified cyt bo_3 was determined *via* Soret Band at 409 nm (Nanodrop DeNovix DS-11) using extinction coefficient value $\varepsilon_{408\ nm}$ = 188 $mM^{-1}\ cm^{-1}$. [2]

Vesicles preparation. Hybrid vesicles were prepared as previously described.[3] Dry *E. coli* polar lipids extract (Avanti Polar Lipids) and block-copolymer poly(butadiene-*b*-ethylene oxide) (PDB_{22}-*b*-PEO_{14}; Polymer Source, P9089-BdEO) were solubilized in chloroform and mixed in glass vials at 1:1 molar ratio. The solution was then dried in a vacuumed desiccator for 2 h to give a thin lipid-copolymer film at the bottom of the glass vial. The lipid-copolymer film was resuspended in 20 mM 4-(2-hydroxyethyl)-1-piperazineethanesulfonic acid (HEPES), 10 mM NaCl buffer (pH7.4) via repeated incubation at 50 °C for 5 min and vortex for 1 min followed by five freeze-thaw-vortex cycles and subsequently extruded 11 times through a 100 nm pore size polycarbonate membrane filter using an Avanti Mini-Extruder to form nanovesicles.

Liposomes were prepared by a similar procedure.[4] 5 mg *E. coli* lipids polar extract (Avanti Polar Lipids) was resuspended in 40 mM 3-(N-morpholino)propanesulfonic acid (MOPS), 60 mM K_2SO_4 (pH 7.0) to a concentration of 20 mg/mL. The suspension was sonicated for 15 s on /45 s off for 6 cycles, and flash frozen and thawed with liquid nitrogen three times. The liposomes were then extruded as described for the hybrid vesicles.

SMA-copolymer-vesicles stability studies. SMA-copolymer was added to achieve a final concentration of 0.5 mM, 1.0 mM, 1.5 mM to separate aliquots of HVs or liposomes at 1.0 mM of total PDB_{22}-*b*-PEO_{14} polymer and lipids mixture. Stability of the vesicles was assessed following 4 h incubation at RT with gentle shaking. The samples were analyzed using DLS (see methods below).

Vesicles destabilization studies. Vesicles (0.94 µmol of total PDB_{22}-*b*-PEO_{14} polymer and lipids mixture) were suspended in a total volume of 1.0 mL. The optical density was measured at 540 nm (OD_{540nm}) using a Shimadzu UV-2450 UV-Vis spectrophotometer. Sequential small volumes containing 0.15 µmol of SMA or Triton X-100 were added to the cuvettes and mixed thoroughly, and the OD_{540}nm was recorded.

Incorporation of $SMA_{cyt\ bo3}$ into vesicles. $SMA_{cyt\ bo3}$ and vesicles, either hybrid vesicles or liposomes, were incubated on ice for 30 min at a protein to lipids content ratio (w/w) of ~1:100 (2:8 protein mass to polymer and lipids mass ratio for SMA_{MPs} directly incubated with HVs). The temperature used for SMA-protein reconstitution in HVs is well above the phase transition temperature (Tg) of the PBd_{22}-*b*-PEO_{14} [Tg = –22 °C according to the supplier Polymer Source]. $MgCl_2$ was added to a concentration of 10 mM and incubated with gentle shaking overnight at 4 °C. The samples were then spun at 17,000 *g* for 15 min, and the supernatant taken forward for other experiments. The concentration of reconstituted protein was analyzed by solubilizing the vesicles in 0.3 % Triton X-100, incubated for 30 min at RT with shaking, and measuring the absorbance band at 409 nm.

Protein reconstitution from DDM. $DDM_{cyt\ bo3}$ was reconstituted as described by Khan *et al*.[3] The vesicles (0.5 mL of 10 mg/mL total lipids and copolymer), were titrated with 10% triton X-100 to 5 x 1 µL aliquots beyond R_{sat}, as indicated by absorbance at 540 nm. $DDM_{cyt\ bo3}$ was added in a protein to lipids ratio of ~ 1:100 (w/w), followed by incubation for 15 min at 4 °C. Four rocker incubations at 4 °C with 30 mg Biobeads were used to remove the detergent (1

h, 2 h, 16 h, and 2 h). The vesicles were then stored at 4 °C. The vesicles were spun at 17,000 *g* for 20 min. Reconstituted protein concentration was analyzed by solubilizing the vesicles in 0.3 % Triton X-100, incubating for 30 min at RT with shaking, and measuring the absorbance band at 409 nm.

Dynamic Light Scattering. Vesicle size distributions were characterized using a Zetasizer Nano ZSP (Malvern Instruments). Samples were equilibrated at 25 °C for 90 s, and a refractive index of 1.45 was used for the hybrid vesicles and 1.37 for the liposomes.[3]

Stepped Mg^{2+} addition to vesicles. 15 µg of $SMA_{cyt\ bo3}$ were incubated with liposomes (1.5 mg/mL of lipids), hybrid vesicles (1.5 mg/mL of total lipids and copolymer) or vesicles-free MOPS buffer (100 µl) on ice for 30 min. $MgCl_2$ was added to a concentration of 1 mM, 2 mM, 3 mM, 4 mM, 5 mM and 10 mM, with incubation at 4 °C and gentle shaking for 2 h. The samples were then spun at 17,000 *g* for 15 min, and the supernatants were collected for analysis via SDS-PAGE.

Clark electrode oxygen consumption measurement. A 0.5 mL buffer of 50 mM potassium phosphate buffer (pH 7) was equilibrated on a Clark-type electrode (Hansatech) at room temperature (-0.6 V). The oxygen concentration in the solution under ambient conditions was taken to be 250 µM. DTT and UQ_1 were added to concentrations of 2 mM and 200 µM respectively. A known concentration of soluble or reconstituted cyt *bo₃* was added and the enzyme turnover determined from the initial oxygen consumption rate.[5,6] When the protein was solubilized in DDM, the buffer also contained 0.05% DDM. When Mg^{2+} was added, the initial buffer also contained the relevant concentration of $MgCl_2$. All measurements were done while stirring.

Detection of cyt bo_3 proton pumping. Vesicles were prepared as described above with the difference that the lipid-copolymer films were resuspended in 0.5 mL of 20 mM MOPS, 30 mM K_2SO_4 buffer containing 5 mM HPTS. Unencapsulated HPTS was removed by size-exclusion chromatography using a Nap-5 G25 column (GE Healthcare) according to the manufacturer's instructions. The vesicles with encapsulated HPTS were diluted to about ~40 µg/mL lipid in a cuvette and the ratiometric fluorescence was monitored in real-time by recording the emission at 510 nm as a ratio of excitations at 405 and 455 nm using a Horiba Quantamaster 8075 spectrofluorometer. Proton pumping was activated by addition of DTT

and UQ_1 to a final concentration of 2 mM and 200 µM respectively. A calibration curve (ratiometric fluorescence *vs* known pH) of HPTS in 20 mM MOPS, 30 mM K_2SO_4 buffer was used to convert the ratiometric fluorescence data to lumen pH values (Figure S6).

SDS-PAGE gel analysis. For SDS-PAGE gel analysis, trichloroacetic acid in dH_2O (100% w/v) and protein samples were mixed in a 1:4 v/v ratio and incubated for 10 min at 4 °C. The proteins were then precipitated *via* centrifugation at 17,000 g for 5 min. The pellets were washed and centrifuged twice with 200 µL cold acetone. The pellets were then solubilized in 14 µL of dH_2O, and 4 µL of 4X SDS-PAGE sample loading buffer was added. The solutions were incubated for 1 hour at 37 °C and then loaded onto a 15% polyacrylamide gel.

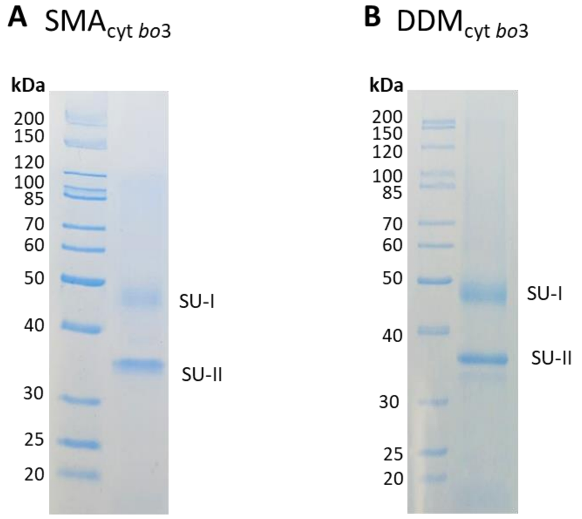

Figure S1. SDS-PAGE (15%) analysis of cyt *bo₃* using two different solubilization approaches. Purified cyt *bo₃* solubilized by SMA (A) or solubilized by DDM (B). Both purification methods were followed by purification *via* Ni^{2+}-NTA-resin column. Protein bands were visualized by Coomassie Blue staining. SU-I = Subunit 1; SU-II = Subunit 2. Subunit 3 and 4 from cytochrome *bo₃* are too small and not visible on these SDS-PAGE.

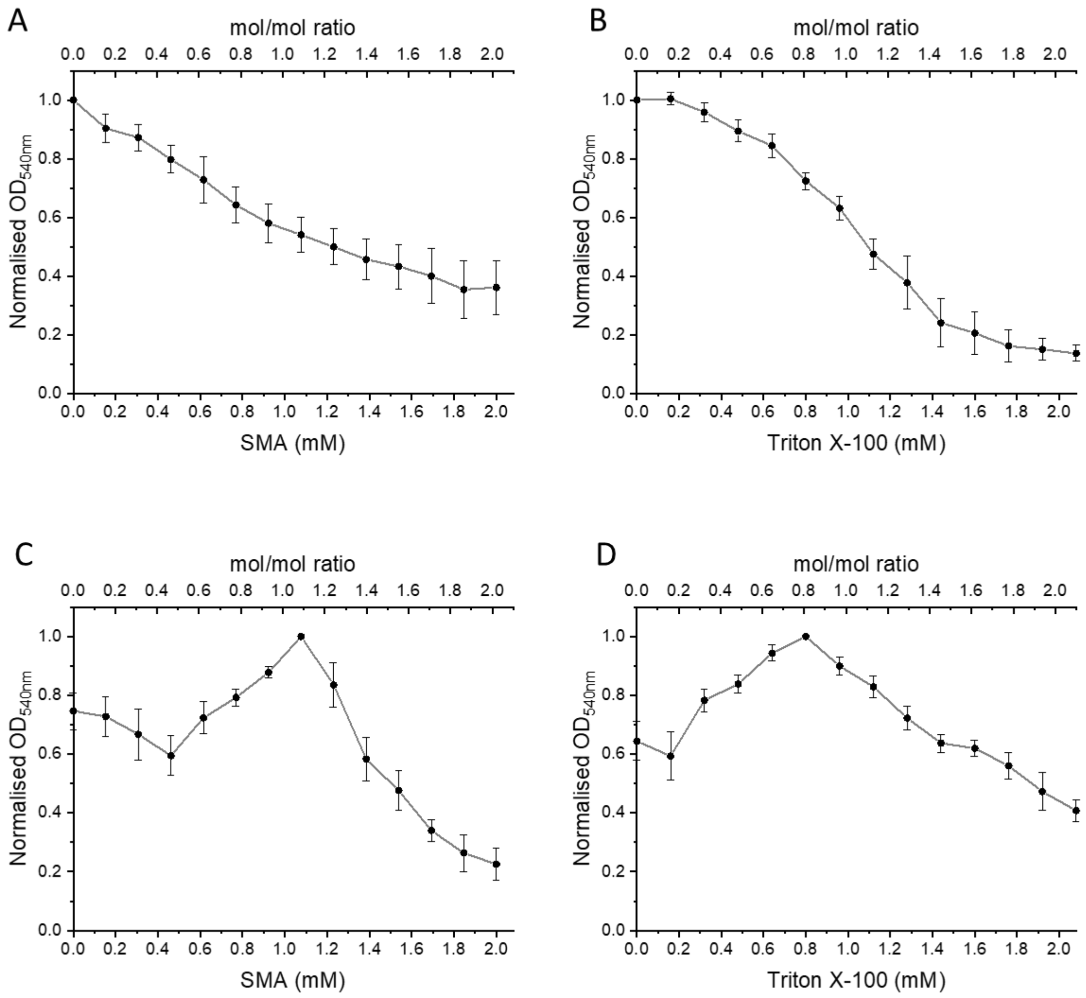

Figure S2. Destabilization profiles of HVs (A and B) and liposomes (C and D) by SMA (A and C) and Triton X-100 (B and D). Absorbance (optical density (OD)) of vesicles was measured at 540 nm in a 1.0 cm path length quartz cuvette.

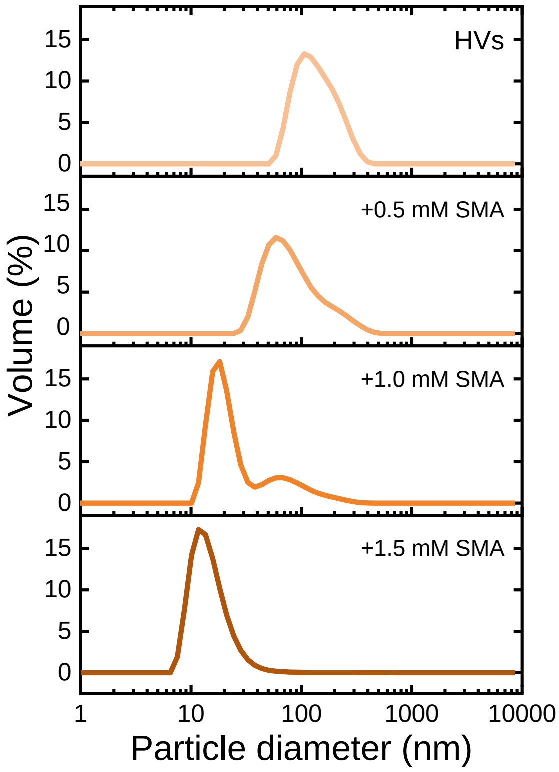

Figure S3. Dynamic light scattering (DLS) volume profiles of HVs (at 1.0 mM of total PDB_{22}-*b*-PEO_{14} polymer and lipids mixture) titrated with increasing SMA copolymer concentration.

Table S1. DLS data for the size distributions shown in Figure 2. Reported are Z-average particle diameter in nm (d/nm), Polydispersity Index (PdI), number of peaks detected, peak size (d/ nm) from the particle size distribution profile by volume and corresponding % and Standard Deviation for a set of representative samples.

**Sample**	**Z-Average (d/nm)**	**PdI**	**Number of peaks**	**Peak size (d/nm)**	**% Volume**	**St Dev (d/nm)**
HVs	129.5	0.088	1	132.00	100.0	54.03
HV-$SMA_{cyt\ bo3}$	149.9	0.118	1	158.8	100.0	72.42
HV-$DDM_{cyt\ bo3}$	97.63	0.233	1	73.82	100.0	62.84
Liposomes	140.3	0.076	1	141.7	100.0	52.26
LIP-$SMA_{cyt\ bo3}$	127.5	0.177	1	128.4	100.0	80.27
LIP-$DDM_{cyt\ bo3}$	116.5	0.219	1	136.2	100.0	131.5

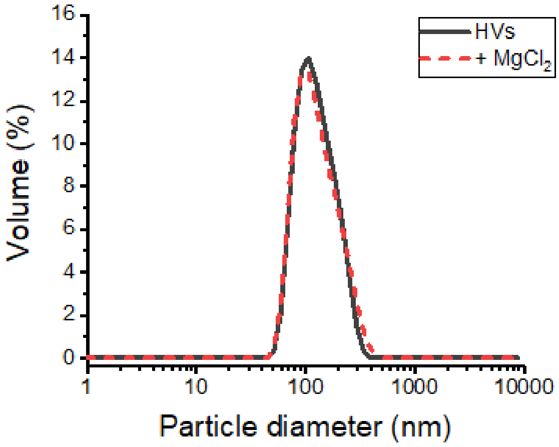

Figure S4. Dynamic light scattering (DLS) volume profiles of HVs before and after treatment with $MgCl_2$ (10 mM).

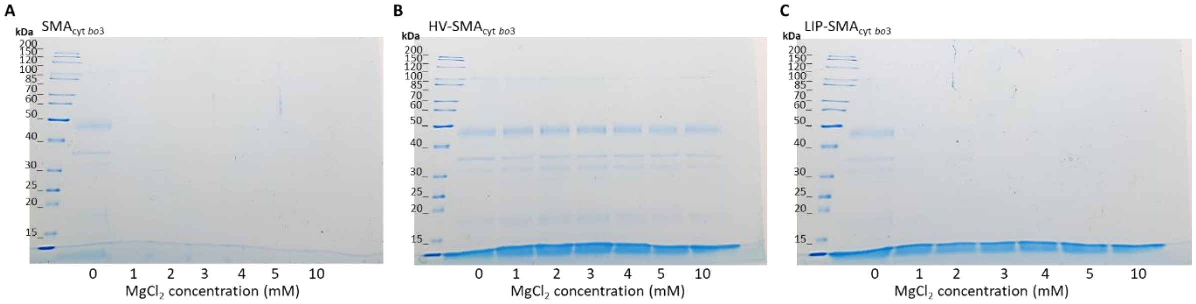

Figure S5. Entire gels for the SDS-PAGE analysis of (A) SMA$_{cyt\ bo3}$ and reconstituted cyt *bo$_3$* in (B) HVs or (C) liposomes shown in Figure 3. After direct incubation of SMA$_{cyt\ bo3}$ with HVs or liposomes, all samples were incubated with increasing Mg^{2+} concentration for 2 h, followed by centrifugation at 17,000 *g* for 15 min to pellet non-reconstituted SMA$_{cyt\ bo3}$. The supernatants were analyzed with SDS-PAGE (Coomassie Blue staining).

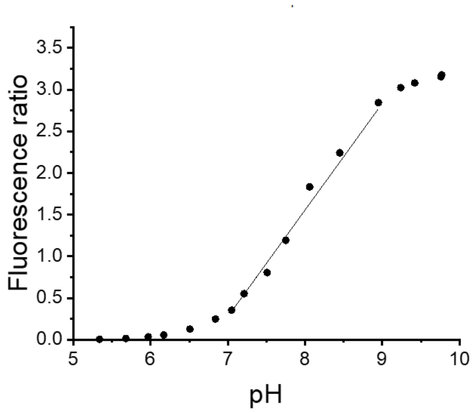

Figure S6. Standard curve for HPTS in 20 mM MOPS, 30 mM K_2SO_4 buffer. For construction of the calibration curve, the pH of the buffer was adjusted in the pH range of 5–10 by sequential addition of aq NaOH or aq HCl until the desired pH was reached. As the pH for these experiments is in the linear range of the calibration curve, only the linear section of the sigmoidal calibration curve was used to convert the HPTS fluorescence ratio to intravesicular pH.

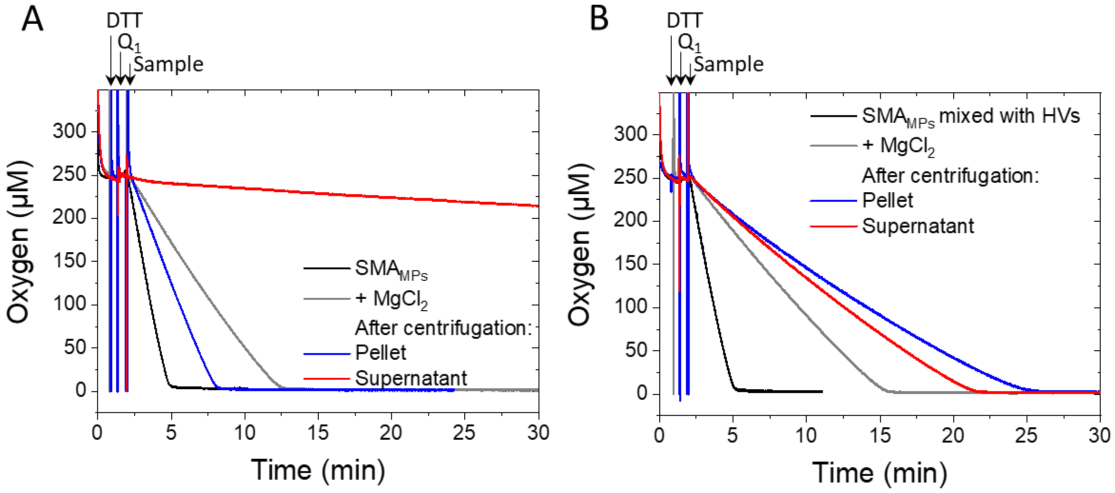

Figure S7. Oxygen consumption traces for cyt *bo*$_3$ activity in (A) SMA$_{MPs}$ and (B) HV-SMA$_{MPs}$ before and after treatment with $MgCl_2$ to remove soluble SMA and SMA$_{MPs}$. Traces are representatives of three independent experiments.

[1] J. N. Rumbley, E. F. Nickels, R. B. Gennis, *Biochim. Biophys. Acta - Protein Struct. Mol. Enzymol.* **1997**, *1340*, 131–142.

[2] J. P. Osborne, N. J. Cosper, C. M. V. Stälhandske, R. A. Scott, J. O. Alben, R. B. Gennis, *Biochemistry* **1999**, *38*, 4526–4532.

[3] S. Khan, M. Li, S. P. Muench, L. J. C. Jeuken, P. A. Beales, *Chem. Commun.* **2016**, *52*, 11020–23.

[4] E. R. Geertsma, N. A. B. N. Mahmood, G. K. Schuurman-Wolters, B. Poolman, *Nat. Protoc.* **2008**, *3*, 256–266.

[5] S. K. Choi, L. Schurig-Briccio, Z. Ding, S. Hong, C. Sun, R. B. Gennis, *J. Am. Chem. Soc.* **2017**, *139*, 8346–8354.

[6] L. L. Yap, M. T. Lin, H. Ouyang, R. I. Samoilova, S. A. Dikanov, R. B. Gennis, *BBA - Bioenerg.* **2010**, *1797*, 1924–1932.
